# Pediatric Area Postrema Cavernoma: Clinical Presentation With Hiccups and Vomiting

**DOI:** 10.7759/cureus.105018

**Published:** 2026-03-11

**Authors:** Amine Kaake

**Affiliations:** 1 Pediatric Neurology, Hospital Robert Debré Ap-Hp, Paris, FRA

**Keywords:** area postrema syndrome, brainstem cavernoma, cerebra cavernous malformation, intractable nausea vomiting hiccups, neurology and pediatric neurology

## Abstract

Cavernous malformations are vascular lesions that may occur throughout the central nervous system. Their occurrence in the medulla oblongata, particularly in the area postrema, is exceedingly rare. Given the role of the area postrema in emesis, lesions in this region may produce a distinctive clinical presentation dominated by vegetative symptoms, such as intractable hiccups and vomiting. Surgical excision can lead to rapid symptom relief but may leave residual neurological deficits due to the eloquence of the brainstem region.

We report the case of a 16-year-old boy with acute onset of neck pain, occipital headaches, paresthesias of the lower limbs, persistent vomiting, and intractable hiccups. Brain magnetic resonance imaging (MRI) revealed an intra-axial lesion within the area postrema, slightly lateralized to the left, with hemorrhagic remodeling suggestive of a cavernous malformation. The patient underwent successful surgical resection, which confirmed the diagnosis. Postoperatively, the hiccups and vomiting resolved completely, although residual left-sided hypoesthesia persisted at three months.

This case illustrates the importance of considering structural lesions, including cavernomas, in the differential diagnosis of intractable hiccups and vomiting. Early imaging and surgical management are crucial to achieve symptom relief and prevent further neurological deterioration.

## Introduction

The area postrema (AP) is a circumventricular organ located on the dorsal surface of the medulla oblongata, beneath the floor of the fourth ventricle. It is part of the dorsal vagal complex, together with the nucleus tractus solitarius and the dorsal motor nucleus of the vagus nerve, and serves as a chemoreceptor trigger zone for vomiting. Its lack of a blood-brain barrier enables the detection of circulating emetic toxins, positioning it as a key integrator of visceral and vegetative responses [1].

Cavernous malformations (cavernomas) are vascular lesions composed of abnormally dilated, thin-walled vessels arranged in a mulberry-like configuration. Their prevalence in the general population is estimated at 0.4-0.5% [2]. While most are asymptomatic, they may present with seizures, focal neurological deficits, or hemorrhage. Brainstem cavernomas are relatively uncommon, representing approximately 9-35% of infratentorial cavernomas [3].

Cavernous malformations in pediatric populations may exhibit a more aggressive natural history compared to adults, with a potentially higher annual hemorrhage risk and cumulative lifetime risk due to longer life expectancy. In children, localization of cavernomas in the brainstem is particularly concerning given the risk of recurrent bleeding and progressive deficits. Despite this, reports of cavernomas specifically involving the area postrema in pediatric patients remain exceptionally rare, with only isolated cases described in the literature [2-4]. This underscores the clinical relevance of the present case.

We report an exceptionally rare pediatric case of area postrema cavernoma presenting with intractable hiccups and vomiting, highlighting the importance of recognizing structural brainstem pathology in children with persistent vegetative symptoms.

## Case presentation

A 16-year-old boy with no relevant past medical history presented in June 2021 with acute neck pain and occipital headache. Within 6 hours, he experienced ascending paresthesias predominantly affecting the left lower limb. Persistent vomiting began shortly thereafter and was followed by continuous hiccups refractory to antiemetic therapy.

At the emergency department, he was hemodynamically stable. Neurological examination revealed ascending paresthesias in the lower limbs, predominantly on the left side, with reduced epicritic and proprioceptive sensation on the left hemibody. No motor deficit was observed, but vomiting and hiccups persisted. No dysphagia, dysarthria, diplopia, or facial sensory deficit was noted. Cranial nerve examination was otherwise normal.

**Figure 1:**
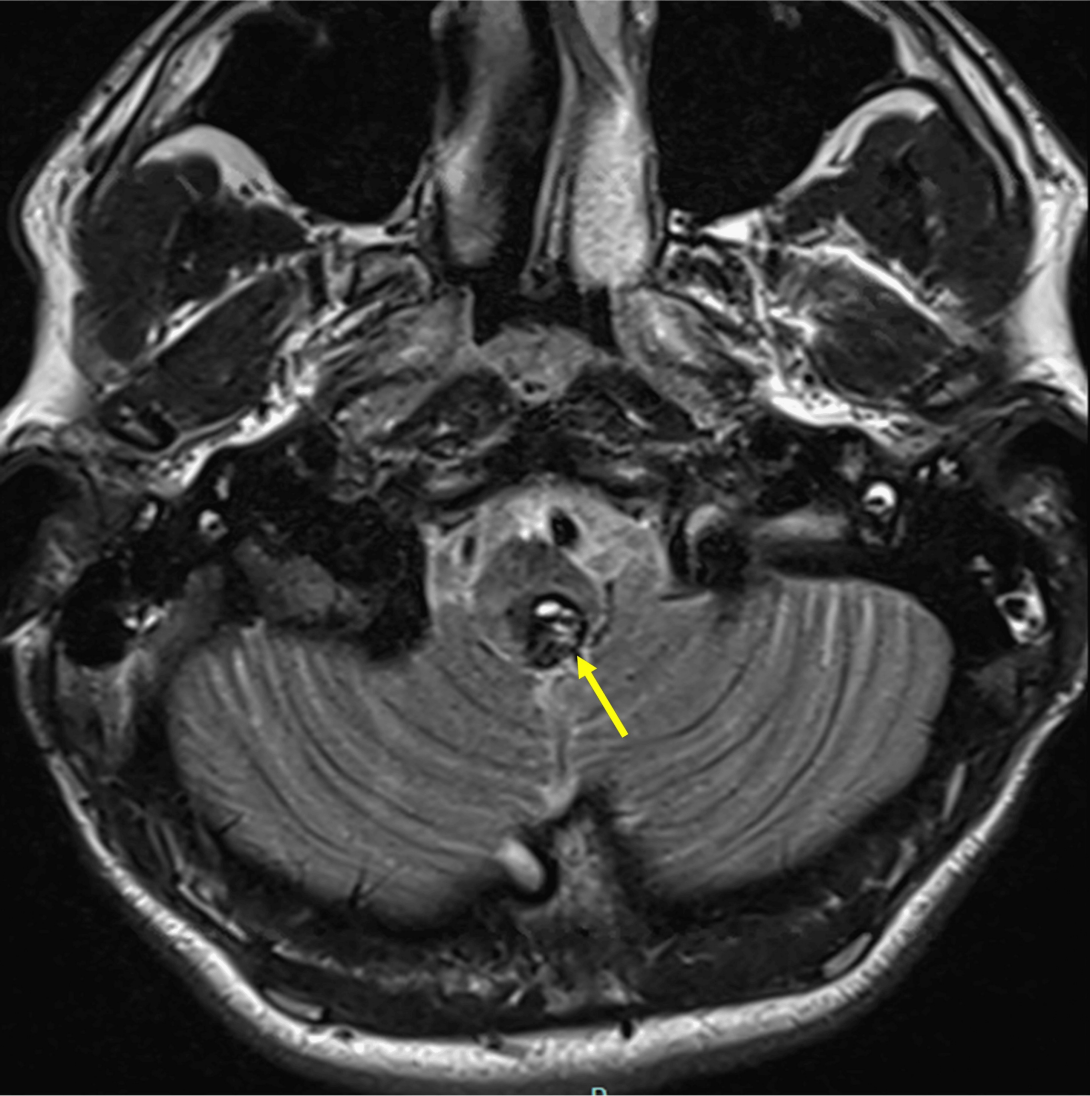
MRI Axial T2 Slice Axial T2-weighted MRI showing a heterogeneous lesion in the dorsal medulla at the level of the area postrema (arrow), with mixed signal intensity and surrounding edema. MRI: Magnetic resonance imaging

**Figure 2:**
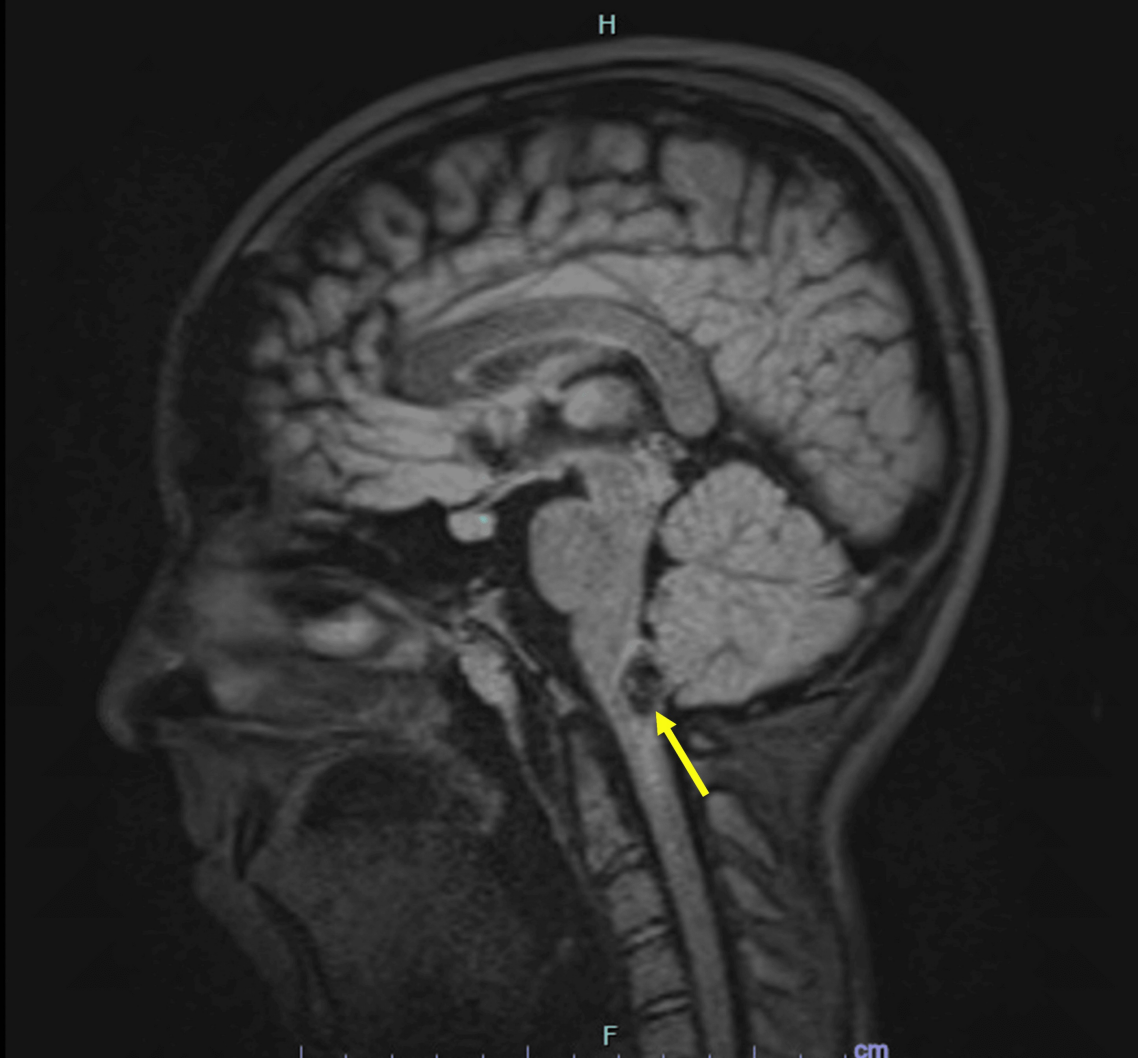
Sagittal T1-weighted MRI demonstrating a nodular intra-axial lesion of the dorsal medulla with intrinsic hyperintensity consistent with subacute hemorrhage (arrow), suggestive of cavernous malformation. MRI: Magnetic resonance imaging

Magnetic resonance imaging (MRI) of the brain showed an intra-axial lesion within the AP of the medulla oblongata, slightly lateralized to the left, measuring 12 × 9 × 14 mm. The lesion appeared heterogeneous, with well-defined borders and hemorrhagic remodeling (T1 hyperintensity), consistent with a cavernous malformation. Blood tests were within normal limits (Figure 1, 2).

Differential diagnoses initially included demyelinating disorders, such as neuromyelitis optica spectrum disorder (NMOSD), acute brainstem encephalitis, and hemorrhagic vascular malformation. The presence of T1 hyperintensity and characteristic MRI features favored hemorrhagic cavernoma. Corticosteroids were administered empirically due to the initial consideration of inflammatory pathology before definitive surgical management.

Given the persistence of sensory symptoms, the patient underwent intravenous methylprednisolone for five days followed by surgical excision of the lesion. The patient underwent microsurgical resection via a midline suboccipital craniotomy with a telovelar approach to the fourth ventricle. Neuronavigation was used to optimize trajectory planning and minimize manipulation of adjacent eloquent structures. Intraoperative neurophysiological monitoring included somatosensory evoked potentials (SSEPs) and motor evoked potentials (MEPs). The lesion was identified at the dorsal medullary surface, corresponding to the area postrema, and carefully dissected from the surrounding neural tissue. Gross total resection was achieved under high magnification.

**Figure 3:**
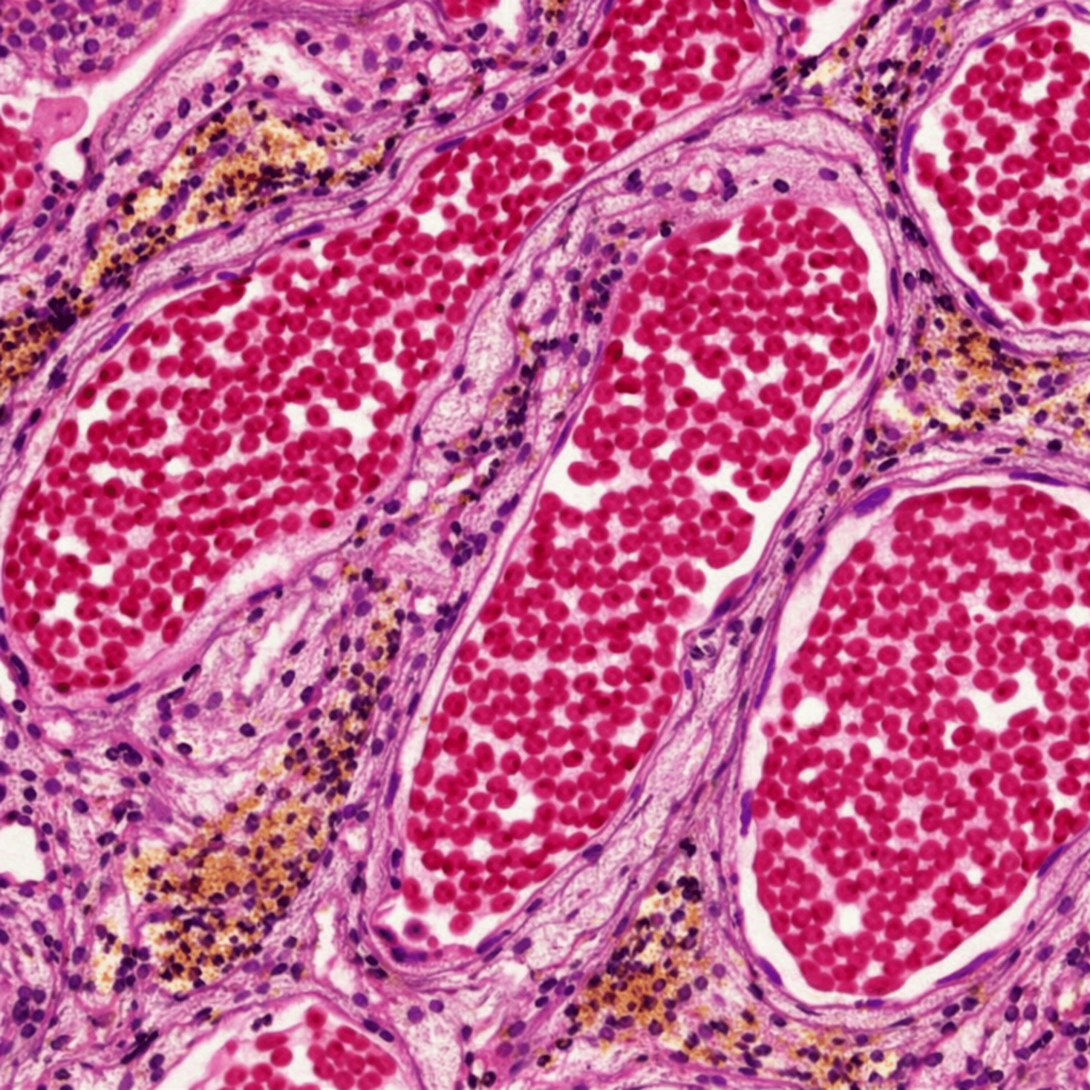
Histopathological confirmation of cavernous malformation Hematoxylin and eosin (H&E) staining demonstrating multiple dilated, thin-walled vascular channels filled with erythrocytes and lined by a single layer of endothelium, without intervening neural parenchyma. Perivascular hemosiderin deposits are also visible, consistent with cavernous malformation.

Gross appearance was suggestive of a cavernous malformation, which was confirmed by histopathological analysis (Figure 3). Postoperative CT revealed no hematoma or unexpected postoperative changes.

Clinically, the hiccups and vomiting resolved immediately after surgery. At postoperative day 7, the patient had mild proprioceptive disturbance and heaviness in the left arm, but motor function was preserved. At three months, he remained with a partial left-sided hypoesthesia with preserved motor function (modified Rankin Scale score: 1) [5], requiring neurorehabilitation. The patient returned to school activities with mild residual sensory deficit.

A postoperative MRI performed three months after surgery confirmed complete resection without evidence of residual lesion.

## Discussion

Brainstem cavernous malformations represent a distinct clinical entity due to their eloquent location and elevated risk of symptomatic hemorrhage. Although they account for approximately 9-35% of infratentorial cavernomas [3], medullary localization is significantly less common, and involvement of the AP remains exceptionally rare. Only isolated cases describing AP cavernomas have been reported, particularly in pediatric patients [4,6,7].

The area postrema plays a central role in emetic regulation as part of the dorsal vagal complex [1]. Its lack of a blood-brain barrier renders it particularly sensitive to circulating toxins and metabolic disturbances. Structural lesions affecting this region may therefore produce a distinctive syndrome characterized by intractable hiccups, nausea, and vomiting. This clinical pattern has been most extensively described in NMOSD, where autoimmune-mediated inflammation of the AP results in the so-called “area postrema syndrome” [8]. However, our case highlights that vascular lesions such as cavernous malformations may mimic this presentation and should be considered in the differential diagnosis, particularly when symptoms are accompanied by focal neurological findings.

In pediatric populations, cavernous malformations may exhibit a different clinical trajectory compared to adults. Although reports remain limited, some studies suggest a higher cumulative hemorrhage risk over a lifetime and a potentially more aggressive course in children, particularly for brainstem lesions [3]. Given the long life expectancy of pediatric patients, even a single hemorrhagic event may justify closer surveillance and lower thresholds for intervention in selected cases. The rarity of reported pediatric AP cavernomas further underscores the importance of documenting such cases to better characterize their natural history and optimal management strategies.

MRI remains the diagnostic modality of choice. The characteristic “popcorn-like” appearance with mixed signal intensity and hemosiderin rim is highly suggestive of cavernous malformation [2]. In our case, T1 hyperintensity indicated subacute hemorrhagic remodeling, supporting the diagnosis. The dorsal medullary location adjacent to the fourth ventricular floor correlated anatomically with the patient’s vegetative symptoms and lateralized sensory findings.

The management of brainstem cavernomas remains controversial. Conservative treatment may be considered in minimally symptomatic patients. However, progressive neurological deficits, recurrent hemorrhage, or disabling symptoms often prompt surgical consideration [4,6,7]. The timing of surgery is particularly debated. Some advocate delayed intervention after hematoma stabilization to reduce operative morbidity, whereas others support early surgery in cases of clinical deterioration [9,10]. In our patient, the persistence of disabling vegetative symptoms and progressive sensory impairment favored early surgical resection.

Microsurgical resection via a telovelar approach allowed direct access to the dorsal medulla while minimizing cerebellar retraction. The use of neuronavigation and intraoperative neurophysiological monitoring contributed to safe lesion removal. Although vegetative symptoms resolved immediately after surgery, mild residual sensory deficit persisted at three months. This outcome illustrates both the potential benefit and the inherent risk of surgical intervention in eloquent brainstem structures [9,10].

Importantly, this case reinforces a key clinical message: persistent hiccups and vomiting, particularly when refractory to medical therapy and associated with focal neurological signs, should prompt early neuroimaging. While inflammatory etiologies such as NMOSD are well-recognized causes of area postrema syndrome [8], structural lesions must not be overlooked. Early identification may facilitate timely management and prevent further neurological deterioration.

## Conclusions

Cavernomas of the AP are exceptionally rare but may present with highly characteristic vegetative symptoms such as intractable hiccups and vomiting. Recognition of this clinical pattern is critical to avoid delays in diagnosis. MRI is essential for identification. In selected symptomatic cases, surgical management may provide rapid relief of vegetative symptoms. However, neurological sequelae can persist due to the eloquence of the brainstem. This case highlights the need for clinicians to consider structural brainstem lesions in the evaluation of persistent vegetative symptoms in children and adolescents.
